# Asthma

**Published:** 1899-01

**Authors:** H. B. Hill

**Affiliations:** Austin, Texas


### TEXAS MEDICAL JOURNAL.

ESTABLISHED JULY, 1885.

PUBLISHED MONTHLY.—SUBSCRIPTION $1.00 A YEAR.

VOL. XIV. AUSTIN, JANUARY, 1899. No. 7.

### Original Contributions.

For Texas Medical Journal.

### Asthma.

BY H. B. HILL, M. D., AUSTIN, TEXAS.

Read at meeting Austin District Medical Society.

This paper will only consider bronchial asthma, and will not include hay-fever, or, as it is sometimes termed, "hay-asthma."

Asthma is a neurosis, the chief characteristic of which is a spasmodic dyspnœa, lasting usually a few hours, but in some cases continuing for several days, or even weeks.

A peculiar condition of the nervous system is the main factor in its causation; but in the way of immediate exciting causes, there is no other disease of which I have any knowledge that is characterized by so much eccentricity. The condition of climate that will cause it in one person will cure it in another. For instance, one person does better at the sea shore, another in the mountains; some in the North, others in the South, etc. This eccentricity is sometimes even manifested in different rooms in the same house.

Another feature that places this disease in the category of medical curiosities, is the great variety of substances or conditions that act as exciting causes. Among the causes, both remote and immediate, may be mentioned (after again referring to the neurotic condition) heredity, pressure of enlarged bronchial glands, rheumatic diathesis, digestive disturbances, nasal obstructions, emanations from a great variety of things, mental or moral impressions, etc. Also that form known as bronchitic asthma seems to be due, to a large extent, to catarrhal causes.

The true pathology of asthma is still shrouded in obscurity. Many theories have been advanced and facts noted, but as yet, none that fully and satisfactorily explain all the phenomena. The limit of this paper precludes entering at length into these various theories. They are referred to in most of the recent text-books. Probably Trousseau had as clear a conception of its pathology as the experimenters of more recent date. Their results seem to confirm his views. A quotation from Stewart and Gibson will show the present status of the question as clearly as can be stated.

"Asthma consists of derangement of the innervation of the bronchial muscles, resulting in irregular and spasmodic contraction of these muscles; and such derangement may consist in an irritability or modification of the respiratory centers, or in irritations applied to their afferent or efferent nerves. Asthma may therefore be purely central in origin, or may have exciting causes in any peripheral part which is capable directly or reflexly of influencing the center of respiration."

The diagnosis of asthma is very easy. The sudden onset, labored breathing, prolonged expiration, clear resonance, and sibilant and sonorous rales, with the absence of the characteristic signs of other forms of dyspnœa, make it evident. When there is cardiac or respiratory disease resulting from or associated with asthma, some difficulty in diagnosing the case may occur. The change of rhythm in respiration, and pearly sputum, are very marked features.

The prognosis is not favorable in most cases as to permanent cure, and although death almost never results directly from attacks of asthma, its tendency to induce disease in several of the organs, usually causes asthmatic subjects to be considered unfavorable risks for life insurance.

We now reach the most important part of the subject, namely: the treatment. There is probably no disease for which a greater number of remedies have been suggested, and none in which a greater number at times are successful. The marked success that has attended such a large variety of remedies, and the neurotic character of the affection, has convinced me that hypnotic suggestion had much to do with the result. Indeed, from its very nature, asthma ought to be adapted to the application of hypnotism. Recent literature reports its frequent successful use in asthma. The efficacy of medicines administered is greatly increased, I am sure, if accompanied by a positive assurance or suggestion that it will give relief in a few minutes.

The object in treatment is to relieve the attack, and in the interval to institute treatment to prevent, as far as possible, a recurrence. The principal advance made in treatment is perhaps not so much in the discovery of new remedies, as the abandonment of such emetic and depressing remedies as lobelia, tobacco, ipecac, tartar-emetic, and blood letting. I will refer briefly to some of the older remedies, and then note some of the later.

The fumes of burning nitrate of potassium, either alone or in combination with stramonium, belladonna, etc., is frequently used with success, and this constitutes the basis of most of the advertised asthma cures.

Next may be mentioned grindelia robusta, cannabis indica, hyoscyamus, ether and chloroform internally or by inhalation, chloral, potass. iodide, and opium. Many others have, from time to time, been recommended, but this list embraces those most frequently of benefit in relieving attacks.

Among the later remedies, are morphine hypodermatically, antipyrin, acetanilid, amyl nitrite (and its congeners), sodium nitrite and nitroglycerin, pyridin, paraldehyde and electricity.

Probably the most efficacious of all remedies is morphine hypodermics, but they do not always relieve, and there are obvious objections to the practice. Chloral, either alone or combined with iodide of potassium, I have seen give very prompt relief in some cases. I have sometimes secured quicker results from amyl nitrite by inhalation than from other methods, but at other times it has only given partial relief from the asthma, and at the same time caused such severe head-ache that it seemed to only substitute one evil for another that was worse. I have seen paraldehyde give refreshing sleep in obstinate cases after morphine and other medicines had failed. Where bronchitis is present, iodide of potassium is nearly always indicated, both during the attacks and in the intervals. I have seen some cases that appeared to be permanently cured after this remedy had been continued for some time. It also is beneficial in cases that appear to be due to the rheumatic diathesis. Some patients have idiosyncrasies that prevent its administration. In such cases, sometimes the syrup of hydriodic acid is better tolerated, and equally useful, but even it cannot be taken by some. Many cases are either cured or much benefited by change of climate, but the climate that is best adapted to each individual has to be determined by experience.

Errors of diet are to be avoided, and digestive disturbances corrected; but the strict low regimen laid down by some authorities is probably neither essential nor beneficial.

Complications and associated diseases should receive appropriate treatment, and the general health should be placed on the best possible basis.

